# Consensus Statement on High-Intensity Focused Ultrasound for Functional Neurosurgery in Switzerland

**DOI:** 10.3389/fneur.2021.722762

**Published:** 2021-09-22

**Authors:** Lennart H. Stieglitz, Markus F. Oertel, Ettore A. Accolla, Julien Bally, Roland Bauer, Christian R. Baumann, David Benninger, Stephan Bohlhalter, Fabian Büchele, Stefan Hägele-Link, Georg Kägi, Paul Krack, Marie T. Krüger, Sujitha Mahendran, J. Carsten Möller, Veit Mylius, Tobias Piroth, Beat Werner, Alain Kaelin-Lang

**Affiliations:** ^1^Department of Neurosurgery, University Hospital Zurich, Zurich, Switzerland; ^2^Neurology Unit, Department of Internal Medicine, Hôpital Fribourgeois (HFR)–Cantonal Hospital Fribourg, University of Fribourg, Fribourg, Switzerland; ^3^Department of Neurology, Lausanne University Hospital and University of Lausanne, Lausanne, Switzerland; ^4^Department of Neurology, Geneva University Hospital and University of Geneva, Geneva, Switzerland; ^5^Department of Neurosurgery, Cantonal Hospital Aarau, Aarau, Switzerland; ^6^Department of Neurology, University Hospital Zurich, Zurich, Switzerland; ^7^Neurocenter, Lucerne Cantonal Hospital, University of Zurich, Zurich, Switzerland; ^8^Department of Neurology, Cantonal Hospital St. Gallen, St. Gallen, Switzerland; ^9^Department of Neurology, Inselspital, University Bern, Bern, Switzerland; ^10^Department of Neurosurgery, Cantonal Hospital St. Gallen, St. Gallen, Switzerland; ^11^Parkinson Center, Center for Neurological Rehabilitation, Zihlschlacht, Switzerland; ^12^Department of Neurology, Center for Neurorehabilitation, Valens, Switzerland; ^13^Department of Neurology, Cantonal Hospital Aarau, Aarau, Switzerland; ^14^Center for Magnetic Resonance (MR) Research, University Children's Hospital Zurich, Zurich, Switzerland; ^15^Neurocenter of Southern Switzerland Ente Ospedaliero Cantonale (EOC), Regional Hospital Lugano, Lugano, Switzerland; ^16^Faculty of Biomedical Neurosciences, Università Della Svizzera Italiana, Lugano, Switzerland

**Keywords:** focused ultrasound, HIFUS, MRgHiFUS, consensus, movement disorders, Parkinson, tremor

## Abstract

**Background:** Magnetic resonance-guided high-intensity focused ultrasound (MRgHiFUS) has evolved into a viable ablative treatment option for functional neurosurgery. However, it is not clear yet, how this new technology should be integrated into current and established clinical practice and a consensus should be found about recommended indications, stereotactic targets, patient selection, and outcome measurements.

**Objective:** To sum up and unify current knowledge and clinical experience of Swiss neurological and neurosurgical communities regarding MRgHiFUS interventions for brain disorders to be published as a national consensus paper.

**Methods:** Eighteen experienced neurosurgeons and neurologists practicing in Switzerland in the field of movement disorders and one health physicist representing 15 departments of 12 Swiss clinical centers and 5 medical societies participated in the workshop and contributed to the consensus paper. All experts have experience with current treatment modalities or with MRgHiFUS. They were invited to participate in two workshops and consensus meetings and one online meeting. As part of workshop preparations, a thorough literature review was undertaken and distributed among participants together with a list of relevant discussion topics. Special emphasis was put on current experience and practice, and areas of controversy regarding clinical application of MRgHiFUS for functional neurosurgery.

**Results:** The recommendations addressed lesioning for treatment of brain disorders in general, and with respect to MRgHiFUS indications, stereotactic targets, treatment alternatives, patient selection and management, standardization of reporting and follow-up, and initialization of a national registry for interventional therapies of movement disorders. Good clinical evidence is presently only available for unilateral thalamic lesioning in treating essential tremor or tremor-dominant Parkinson's disease and, to a minor extent, for unilateral subthalamotomy for Parkinson's disease motor features. However, the workgroup unequivocally recommends further exploration and adaptation of MRgHiFUS-based functional lesioning interventions and confirms the need for outcome-based evaluation of these approaches based on a unified registry. MRgHiFUS and DBS should be evaluated by experts familiar with both methods, as they are mutually complementing therapy options to be appreciated for their distinct advantages and potential.

**Conclusion:** This multidisciplinary consensus paper is a representative current recommendation for safe implementation and standardized practice of MRgHiFUS treatments for functional neurosurgery in Switzerland.

## Introduction

Magnetic resonance-guided high-intensity focused ultrasound (MRgHiFUS) provides a novel ablative treatment option currently applied mainly for essential tremor (ET) and to a lesser extent for tremor-dominant Parkinson's disease (PD) (1, 2). However, adoption of MRgHiFUS into clinical standard of care and present revival of ablative interventional therapy in functional neurosurgery raises a host of challenges for the neurosurgical and neurological community. For MRgHiFUS interventions with a relatively solid data basis of clinical experience, standardization of patient selection and assessment, time point for therapy, treatment targets and alternatives, procedural details, patient management before, during and after MRgHiFUS, outcome measurements, and benchmarking are highly needed.

Functional neurosurgical treatment of brain disorders requires multidisciplinary collaboration among various clinical specialties. Apparent elegance of MRgHiFUS technology and simplicity of intervention processes make it easy to forget about the complexity of the pathophysiology of tremor. With the expected sharp rise in MRgHiFUS intervention numbers and given that MRgHiFUS is only at the beginning of its technological maturation curve, it is important that all specialists involved in care and management of patients suffering from neurological and psychiatric disorders have access to up-to-date information about the relevant technical and medical peculiarities of MRgHiFUS-based interventional therapy.

Many issues concerning application of MRgHiFUS in functional neurosurgery remain controversial, whereas currently no clear guidelines regarding MRgHiFUS treatment exist. In order to aggregate and harmonize current clinical knowledge and experience regarding state-of-the art therapy of various movement and pain disorders with a special focus on MRgHiFUS, two complementary workshops and consensus meetings in Zurich as well as one additional online meeting were held, gathering participants from most of the specialized centers for the treatment of movement disorders in Switzerland.

Here we present the consensus statement on clinical application of MRgHiFUS for functional neurosurgery as formulated by the newly established Swiss MRgHiFUS Working Group.

### Current Status

Although focused ultrasound has been applied as a neurosurgical tool for more than 70 years (3), only the recent introduction of modern MRgHiFUS devices finally allowed routine precise and safe ablation of targets located deep inside the brain through the intact skull (4).

The only commercially available transcranial MRgHiFUS system (ExAblate Neuro™; INSIGHTEC Ltd., Tirat Carmel, Israel) consists of a phased array piezoceramic helmet with 1,024 elements that can precisely focus ultrasound waves into circumscribed brain locations. A 1.5 or 3 Tesla MRI scanner (GE Healthcare, Chicago, IL, USA and Siemens Healthineers, Erlangen, Germany) for target verification, real-time thermal feedback and treatment planning, guidance and lesion confirmation complete the MRgHiFUS equipment.

MRgHiFUS has emerged as a neurosurgical option mainly for the therapy of movement disorders (1, 2) but also for pain syndromes (5). The procedure received FDA clearance with MRI scanners from GE Healthcare in the USA for ET and PD in 2016, and CE marking additionally for Siemens Healthineers MRI systems in the European Community for neurological disorders, specifically ET, tremor-dominant PD and neuropathic pain in 2018. Studies on novel indications such as psychiatric diseases, epilepsy and brain tumors, and on blood brain barrier opening for targeted drug delivery against neurodegenerative diseases and brain tumors are underway (6).

In Switzerland, three centers, the University Hospitals Zurich and Geneva, and the SoniModul AG Solothurn, are offering MRgHiFUS interventions in a clinical setting. In Zurich, clinical applications of MRgHiFUS in the brain have been studied since 2006 and, as early as 2009, the first focused ultrasound interventions in the central lateral nucleus of the thalamus for chronic neuropathic pain as a pilot application were reported (7).

### Consensus Meetings and Workshops

#### Invitation

Motivated by the installation of a new clinical treatment system for MRgHiFUS at the University Hospital Zurich and the imminent addition of MRgHiFUS functional neurosurgery interventions into the reimbursement catalog of the Swiss public health system, the Swiss Movement Disorders Society (SMDS) board decided to develop a set of guidelines for the reintroduction of basal ganglia ablative functional neurosurgery in Switzerland with MRgHiFUS technology. The guidelines should build on the experience of movement disorders specialists from classical radiofrequency lesioning, deep brain stimulation (DBS) and MRgHiFUS and accordingly, a workgroup was formed (Swiss MRgHiFUS Working Group) involving neurologists, neurosurgeons and medical physicists from all over Switzerland.

Invitations to participate in the meetings were sent to all involved experts in Switzerland (1) all chairmen of neurological departments of Swiss university hospitals and cantonal hospitals, (2) all heads of movement disorders units in Switzerland, (3) all neurologists and neurosurgeons already performing MRgHiFUS treatments in Switzerland, and (4) all members of the SMDS (see Participants for more details about the invitation process). For workshop preparation, participants received a booklet containing a list of all participants and their affiliations, a comprehensive literature research and overview over the pertinent publications on MRgHiFUS and basic information about MRgHiFUS technology (see Selection of literature for literature selection). The literature was selected by the organizing committee, LS, GK and AK, and comprised the literature cited in this manuscript. Furthermore, they were invited to provide suggestions for key questions and topics to be discussed. The objectives of the meetings and the consensus statement were to provide a specialist framework and guidance to those embarking on MRgHiFUS therapy including patient selection, indication, procedure, aftercare, alternatives, and educational opportunities, to discuss and highlight the potential, challenges, benefits and distinctive risks of MRgHiFUS, and to promote scientific activities and participation into clinical trials and registries.

#### Participants

**Figure 1:**
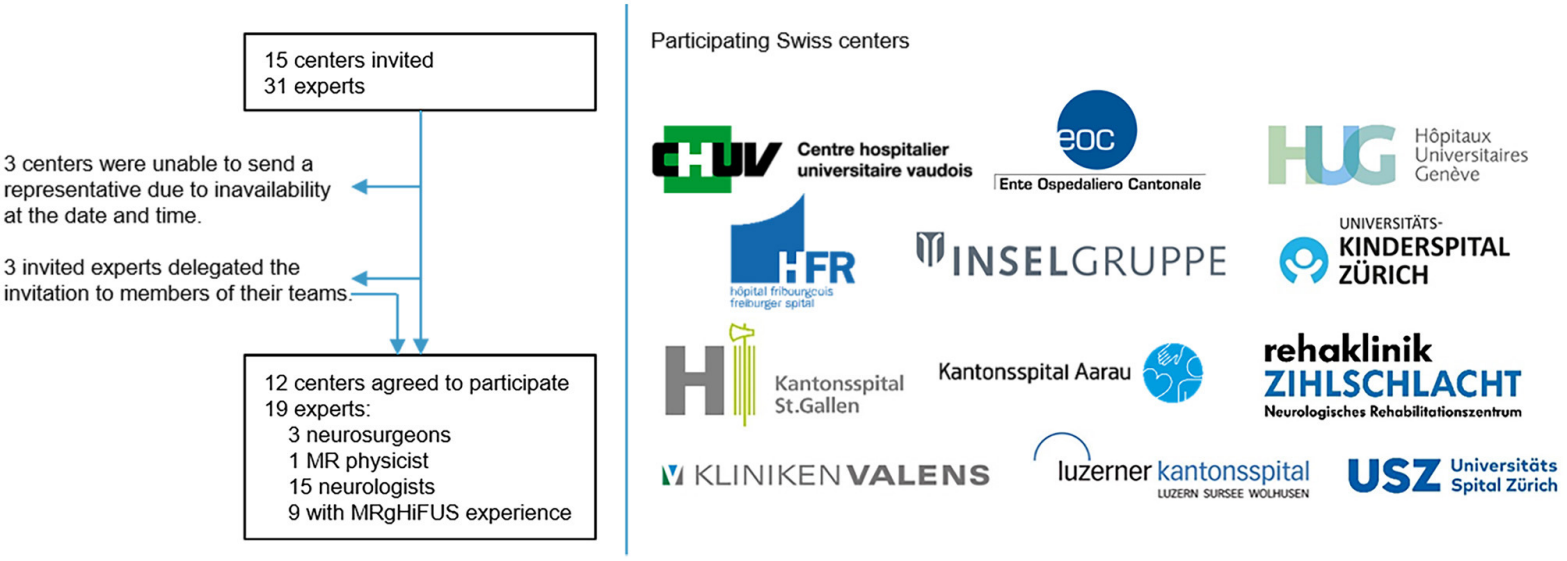
Process of invitation for constitution of the MRgHiFUS workgroup.

All known experts in the field of movement disorders in Switzerland were contacted via direct e-mail by the first author (LS). In total 19 experts from 15 centers and members of 5 societies across Switzerland participated in the two workshops and consensus meetings and the online meeting, comprising 14 neurologists, 4 neurosurgeons and 1 medical physicist. Nine of the participants had personal experience with MRgHiFUS treatment. Summarizing the Hôpital Fribourgeois (HFR), Hôpitaux Universitaires de Genève (HUG), Center Hospitalier Universitaire Vaudois (CHUV), Kantonsspital Aarau (KSA), Universitätsspital Zürich (USZ), Luzerner Kantonsspital (LUKS), Kantonsspital St. Gallen (KSSG), Neurocenter of Southern Switzerland EOC Inselspital Bern, Zentrum für Neurorehabilitation Zihlschlacht, Kliniken Valens, and Kinderspital Zürich, all regions of Switzerland are represented (Figure 1).

#### Selection of Literature

**Figure 2:**
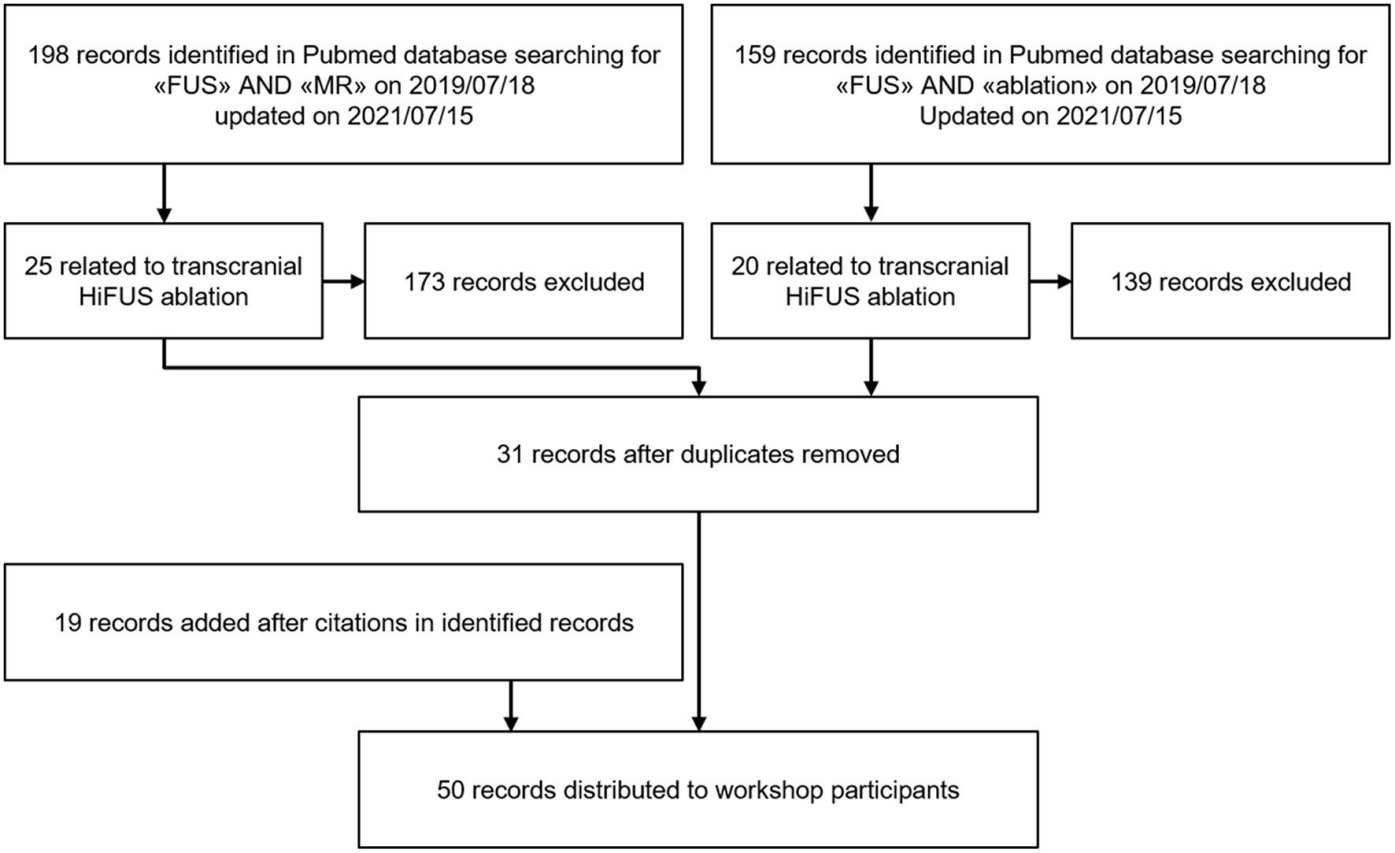
Process of literature research and dissemination to workshop participants.

The selection of the literature followed PRISMA guidelines and is illustrated in Figure 2. The process of literature selection was repeated before the third meeting to cover recent publications, which were not discussed during the first two meetings. Only clinical papers were selected, purely technical and pre-clinical studies were excluded. The search terms were chosen very unspecific (“FUS” and “MR”) to avoid accidental exclusion of relevant literature. All records were viewed by the organizing team (LS, AK and GK) for exclusion of all records not related to transcranial MRgHiFUS treatment. As a last step, all remaining articles were reviewed for citations of relevant articles which were not listed so far.

#### Workshop

The workshops and consensus meetings took place in Zurich on 2.12.2019 and 17.01.2020, respectively, in the form of moderated, interdisciplinary roundtable discussions. A third meeting was held online on 26.07.2021 to comply with the hospital regulations of the Covid-19 pandemic. Meeting minutes were recorded by the first author (LS) and reviewed by all participants.

The first meeting was moderated by a Swiss journalist, former redactor and director of a large Swiss media company and advisor for several Swiss cantonal health directors, who was booked for this occasion by the SMDS board to guarantee a fair and balanced discussion.

### Method

#### Consensus Method

**Figure 3:**
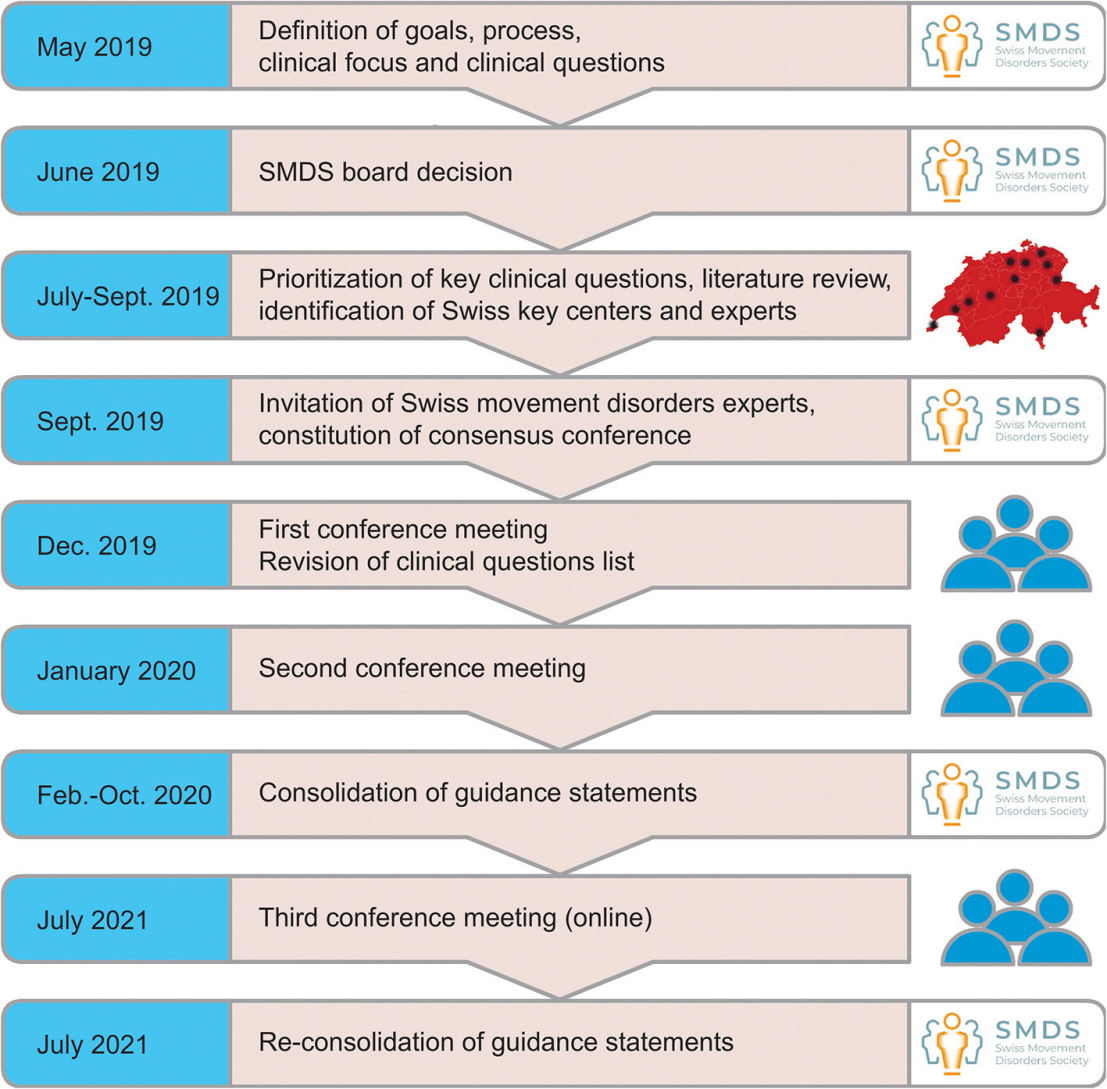
Overview over the process used by the Swiss MRgHiFUS Working Group for definition of topics, consolidation of an expert group and developing consensus on MRgHiFUS treatment in Switzerland.

The process of the consensus meetings is illustrated in Figure 3. The meetings followed a modification of the Nominal group technique (8). The participants were asked to brainstorm in private and provide their results to the organizing team. Those topics were again distributed among the participants before the meeting together with an overview of the relevant literature. During the first two meetings, those topics were again enriched through a second. Afterwards each of the topics was discussed in plenum, followed by open voting (hand-raising) to generate a common statement. The statements were collected by LS and drafted into a statement manuscript, which was again re-read and evaluated by all conference members before clearance for dissemination. As there were many important results published before finalization of the consensus statement, a third conference was held in which the most recent literature was considered and updated after another voting. The evidence level of the expert commission's statements is IV.

#### Topics and Questions

During October 2019 and January 2020, the organizers drafted a meeting agenda including a list of relevant topics and questions to be discussed by the new Swiss MRgHiFUS Working Group. Based on a comprehensive literature review, special emphasis was put on current experience and practice, and areas of controversy regarding clinical application of MRgHiFUS for functional neurosurgery. All participants were asked to provide additional topics for the conference which were disseminated among the group together with the relevant literature in advance.

## Consensus Statement

### Reintroduction of Lesioning

*Should lesioning (irrespective of modality) be considered a viable treatment option for movement disorders despite the success of reversible DBS?*

After the introduction of DBS, Professor Alim-Louis Benabid, one of the pioneers of DBS, claimed he would never perform a lesion again in a diseased brain (personal recollection of a conversation by one of the authors). However, initial clinical success and technological potential of MRgHiFUS warrant reopening a therapy field that was considered closed.

Technically, the innovations distinguishing MRgHiFUS from radio-frequency ablation (RFA) is (i) incisionless delivery of therapy, (ii) intraoperative image guidance, (iii) intraoperative ablation control, and (iv) progressive and better-controlled ablation. Together, they enable a more precise ablation of small functional targets deep in the brain which, owing to improved anatomical and pathophysiological understanding, are much clearer defined than in the initial days of lesioning. Compared with radiosurgery (e.g., Gamma knife, GKRS), thermal lesions created by MRgHiFUS become immediately effective and allow both intra-operative image-based and neurological assessments of induced lesions.

While physiologically thermal lesions created by RFA and MRgHiFUS are similar, they differ in spatial geometry with MRgHiFUS lesions being much sharper delineated than RFA. Therefore, it will be challenging to translate previous clinical experience and literature from RFA interventions toward new MRgHiFUS lesioning. Most importantly, modern image guidance will help reduce adverse effects from mistargeting and will require an unbiased reevaluation of stereotactic targets both in terms of therapeutic benefit and in terms of associated risks.

#### Evidence

The effects of MRgHiFUS on brain tissue are well-known and it could be histologically demonstrated, that an ablative effect is reached beyond a specific threshold over 50°C (9). De Vloo et al. used the opportunity to perform microelectrode recording in a patient who underwent unsuccessful MRgHiFUS treatment reaching only subtherapeutic temperatures. They found normal cell activity and argued, that this finding supports the expectation of not harming brain tissue below a threshold of 55°C (10). Dallapiazza et al. compared the results of DBS, MRgHiFUS, GKRS and RFA in a systematic review and found evidence, that indeed the use different technologies in the same targets leads to comparable results (11).

#### Expert Opinion

The workgroup unequivocally recommends further exploration and adaptation of MRgHiFUS-based functional lesioning interventions as a complement and possible alternative to existing treatment approaches such as DBS and confirms the need for outcome-based standardized evaluation of these approaches based on a unified registry.

### Indications for MRgHiFUS

*For which indications should MRgHiFUS be included into standard of care; which indications should require further evaluation in clinical trials and for which indications should application of MRgHiFUS be discouraged?*

Technically, MRgHiFUS can create small thermal lesions in the thalamus and subthalamic area. Larger targets, or targets that are located deeper or more peripherally, could potentially also be accessed, but might need prolonged treatment times or adapted patient positioning. Obvious candidates for MRgHiFUS treatments are therefore patients with most indications that are currently treated interventionally, especially with DBS:

- tremor (ET and PD tremor) by ventral intermediate thalamic nucleus (VIM) lesioning
- PD with fluctuations and dyskinesias by unilateral subthalamic nucleus (STN) lesioning
- dystonia by bilateral globus pallidus internus (GPi) lesioning
- chronic neuropathic pain by centromedian thalamus (CMT) and central lateral thalamus (CLT) lesioning
- psychiatric disorders such as OCD with anterior limb of internal capsule (ALIC) lesioning

#### ET

##### Evidence

Recent randomized controlled studies (RCTs) have shown that MRgHiFUS treatment works well for ET (2, 12, 13). According to the currently available data (14), unilateral DBS and unilateral MRgHiFUS in the VIM seem to be equally effective in ET. For thalamic DBS, in some cases long-term habituation was reported. The underlying mechanisms are not clear so far (15). For thalamic RFA, habituation was not reported so far (16, 17). Of course, the data available for DBS is far more extensive than that for MRgHiFUS and thus a direct head-to-head comparison is impossible yet. To identify, whether habituation is solely a topic in DBS and not in MRgHiFUS, more evidence is needed.

##### Expert Opinion

Because of its proven effectivity and low complication rates, MRgHiFUS could develop from a second-choice treatment option for patients with contraindication to DBS into a preferred option for a very specific sub-group of patients (class Ia evidence for MRgHiFUS thalamotomy in ET). F.i. patients with only mild tremor severity (partially refractory to medications, disabling from the patient's perspective, but too low to justify the risks of DBS) might be a good choice as well as patients of advanced age (too old for DBS), or patients with very asymmetric tremor involving the dominant hand.

With ET being mainly an isolated disease with typically only low neurological comorbidity, MRgHiFUS could be offered by specialized multidisciplinary interventional treatment centers in an easier way.

#### PD

##### Evidence

Unilateral MRgHiFUS lesioning of VIM for tremor-dominant PD has received FDA clearance in 2018 and seems to be a viable treatment option (18). With published experience from RFA predicting significant risk of side effects for bilateral treatments, bilateral lesioning of the VIM or STN should not be conducted outside RCTs until careful evaluation of outcome data allows separation of risk factors, such as mistargeting or bad target selection from patient-specific, respectively, disease-related causes.

Recently, the effective reduction of motor features in PD with asymmetrical medically refractory motor signs and/or fluctuations by unilateral subthalamotomy using MRgHiFUS technique was shown in a randomized controlled trial with low complication rates, although new-onset dyskinesia during the on- and off-state has been observed in some patients (19). Another approach represents the unilateral pallidotomy in PD patients with L-dopa responsiveness and asymmetrical fluctuations and dyskinesias. Eisenberg et al. published a non-controlled open-label trial in 20 patients with 59% improvement of UDysRS in on-medication state and low complication rates (20).

##### Expert Opinion

Because tremor is only a facet of the complex PD pathology, the further course of the disease must be considered when evaluating patients and selecting ablation targets. Although the first results of subthalamotomy and pallidotomy in PD patients are promising, the selection of patients for unilateral treatment will be of key importance (class Ib evidence for subthalamotomy and pallidotomy in PD). Involvement of a specialized neurological department, offering alternative treatments such as conservative therapy, drug delivery pumps or DBS, is mandatory. Beside neurological, also neuropsychological and neuropsychiatric expertise needs to be integrated, typically in the form of multidisciplinary boards.

#### Dystonia

##### Evidence

For the treatment of dystonia, only a single case of MRgHiFUS ablation in the ventral oral anterior thalamic nucleus (VOA) has been reported so far. Although it might be a potential indication, further studies are needed despite the technical and medical challenges involved in conducting the intervention (21). Most probably, interventions would require patients to be anesthetized to ensure proper immobilization, leaving neurological monitoring to minimal assessment of adverse effects. In addition, MRgHiFUS treatment of GPi may even cause PD-like side effects or even induce a parkinsonian syndrome (22). A conceivable target would be the VOA, especially because of promising data of previous thalamotomies (23, 24). First experiences with pallidotomy in PD patients indicate, that the technical issues might be solvable, and the side effects and complications may be low (20).

##### Expert Opinion

Ablative treatment of dystonia has an interesting potential. The key issue will be the safety of the procedure. Currently, only very little experience exists with MRgHiFUS pallidotomies, therefore the risk/benefit ratio may not be estimated properly. Thus, treatments should only be performed within randomized controlled trials. Based on the paucity of experience so far (class IIb evidence for MRgHiFUS pallidotomy in PD), MRgHiFUS treatment for dystonia is currently not recommended for clinical application.

#### Neuropathic Pain

##### Evidence

Therapy-resistant pain was one of the first indications treated with MRgHiFUS (7). The treatment of chronic neuropathic pain (type 2) by RFA and MRgHiFUS is effective and well-established. Experience with MRgHiFUS for pain is reported from the consortium although few of the Swiss data is systematically evaluated and published so far. Therefore, the currently available data is sparse and insufficient. Different targets are appropriate, among them the CMT or ventroposteromedial thalamic nucleus (VPM) and ventroposterolateral thalamic nucleus (VPL) (5).

Lesioning of the VPM and VPL could be associated with sensory deficits and therefore, the CMT should be preferred. Alternatively, the lateral portion of the cuneocerebellar tract (CCT) could be used.

##### Expert Opinion

No general recommendation can be made for patients suffering from chronic pain. Indications for treatment including MRgHiFUS should be restricted to the confines of RCTs and should be made within the framework of an interdisciplinary pain board.

#### Epilepsy

##### Evidence

Regarding epilepsy therapy and targeting of e.g., the anterior thalamic nucleus (ANT) with MRgHiFUS, no relevant data exists, no experience was reported, and this therapy therefore currently remains widely hypothetical and experimental. Lesioning the ANT might be a promising option in therapy-resistant epilepsy. Still, this would be a completely novel treatment and should only be performed within the confines of a study protocol.

##### Expert Opinion

Beside ET and tremor-dominant PD, MRgHiFUS treatments for movement disorders and other brain disorders in general are still under clinical evaluation or even considered experimental.

#### Obsessive-Compulsive Disorder

##### Evidence

For cases of intractable OCD, DBS as well as ablative neurosurgery have been tried as escalation therapies (25). Among the ablative techniques, only gamma ventral capsulotomy was examined in an RCT with a response in 3/8 patients at 12 months and 5/8 patients at 54 months and no severe complications, indicating that ventral capsulotomy is a viable alternative to DBS in selected cases (26, 27). A recent phase I trial published by Davidson et al. reported a response in 4/6 patients after MRgHiFUS ventral capsulotomy in OCD patients with no serious adverse events (class IIb evidence) (28).

##### Expert Opinion

Treatment of intractable OCD with ventral MRgHiFUS capsulotomy might be efficacious. So far, only very small studies have been published and a sufficiently long follow-up seems to be crucial to correctly evaluate the efficiency of the treatment. Furthermore, ventral capsulotomy must be evaluated against DBS, for which more experience is published from randomized controlled trials. At the given time, treatment of intractable OCD with MRgHiFUS should be restricted to the confines of RCTs.

### Clinical Setting for MRgHiFUS

*In which clinical settings should MRgHiFUS be performed?*

#### Evidence

There is presently no evidence available regarding required clinical settings for MRgHiFUS.

#### Expert Opinion

Therapeutically, MRgHiFUS is an incisionless, ablative procedure involving low but real risks of neurological complications and adverse effects due to unintentional effects to non-targeted functional units, unexpected side effects of network modulation and, most significantly, microscopic and macroscopic bleeding. It is therefore considered essential that MRgHiFUS treatments are conducted under supervision of experienced functional neurosurgeons and specialized neurologists. Furthermore, centers offering MRgHiFUS should have an adequate access to emergency neurosurgery and anesthesiology, and neurointensive or intermediate care. This means that centers offering MRgHiFUS treatment must be able to diagnose and treat emerging complications autonomously by providing permanent availability of experts from different disciplines and professions like neurology, neuroradiology, neuro-intensive care, and physiotherapy. Furthermore, an adequate patient selection requires a multidisciplinary team for the evaluation of the patient before the intervention, including assessment of motor and non-motor symptoms, e.g., cognitive impairment not allowing the adequate communication during MRgHiFUS.

Hence, MRgHiFUS treatment should only be conducted in clinical settings where multiple treatment options can be offered and where therapy options can be evaluated by multidisciplinary movement disorder boards, which usually are located at specialized centers and university hospitals.

### Patient Selection for MRgHiFUS

*Which patient groups could benefit most from MRgHiFUS?*

#### Evidence

So far, all randomized controlled studies on MRgHiFUS selected patients who were in one way or another not suitable for DBS (1, 2).

#### Expert Opinion

During its clinical introduction, MRgHiFUS has been considered an interventional treatment option mainly for patients who presented with contraindications for DBS. Such cases could include patients not tolerating electrode implants, patients not able to adequately handle the DBS devices, or patients with brain pathologies not eligible for insertion of electrodes and DBS. With clinical acceptance of the method, patient selection can start focusing on individual risk-benefit evaluations between MRgHiFUS, DBS and other modalities. Therapeutically, the main difference between MRgHiFUS and DBS-based modulation of neuronal circuits is the permanent nature of thermal lesions, which cannot be altered once they are set, respectively, can only be increased in size if not effective enough. Accordingly, patient selection should focus on patients where post-operative optimization of intervention outcome is not a priority or where the underlying pathology is not expected to require repeated adaptation of network modulation in the near future such as PD. In terms of indications, ET seems a good indication for MRgHiFUS because it mostly does not follow a course of multi-systemic progression such as PD and even improved albeit residual tremor can result in good quality of life (QoL). In terms of patient cohorts, MRgHiFUS could be an attractive option for elderly patients in general, where improving prominent symptoms might provide significantly increased QoL within the relevant time horizon. It should be noted that the lack of post-interventional effect tuning goes together with reduced clinical time and effort and accordingly reduced financial burden for the healthcare system.

### Safety Procedures and Contraindications for MRgHiFUS

*Which safety procedures and contraindications should be respected to ensure maximum patient safety during the procedures?*

#### Technical Aspects

##### Evidence

The efficiency of focusing acoustic energy into a circumscriptive target inside the skull for thermal ablation depends strongly on the acoustic properties of the patient skull which have high inter-individual variability. A rough estimate of expected focusing quality is provided by the skull density ratio (SDR), which is calculated from preoperative computed tomography data of the patient's head. Typical SDR values for Caucasian patients are between 0.45 and 0.80, whereas SDR should not have values below 0.35 for thalamic targets and 0.4 for extra-thalamic targets such as the GPi. Low SDR values imply poor focusing quality, i.e., less precise lesion definition and compensating for associated low focusing gain will require higher acoustic energies, leading to more skull heating with the risk of unintended damage to skull bone-marrow. Ultimately, under-treated stereotactic targets because of insufficient total thermal dose are a risk (29). Pathological deformations of skull shape, skull bone replacements and other artifacts of past surgical interventions may reduce usable skull surface to a degree where inacceptable skull heating might occur.

##### Expert Opinion

Knowing and respecting ultrasound physics and the limits of the technique is essential to prevent possibly harming the patients either by insufficient treatment, which then needs to be repeated with other techniques, or treatment side effects. Thus, MRgHiFUS should only be performed in a center providing the needed technical expertise and support.

#### Patient Cooperation

##### Evidence

To the best of the authors' knowledge, there is no data available regarding the need for cooperation of the patients during MRgHiFUS treatment.

##### Expert Opinion

MRgHiFUS interventions are conducted by repeating sonications with stepwise increased energy levels and resulting peak temperatures at target to allow constant evaluation of intended treatment effects and unintended side effects. Patient cooperation for neurological testing is therefore essential and reliable two-way communication between treatment team and patient is required.

Treatments under general anesthesia would technically be possible, but the lack of neurological testing as a means of target verification might increase the risk for complications due to mistargeting or excessive lesion size. Therefore, such treatments are discouraged for the time being.

#### Patient Medical Status

##### Evidence

The authors are not aware of any published experiences regarding MRgHiFUS under effective anticoagulation or antiaggregation. Microbleeds in the core of the lesion, on the other hand, are regularly observed by neuroimaging (30, 31).

##### Expert Opinion

Although MRgHiFUS does not require a skin incision or skull trepanation, the energy applied to the skull and brain via ultrasound waves is invasive. Associated risks include cavitation effects to blood-vessels and to the margins of intracranial cysts like Virchow-Robin spaces. Consequently, though the risk of intracranial hemorrhage may be low, normal coagulation status and absence of any antiaggregatory or anticoagulative therapy must be assured preoperatively. The risk for convulsive epileptic seizures during the treatment should be low as such an event could endanger the patient when occurring with the head fixed in a stereotactic frame. Furthermore, the risk of severe nausea and vomiting due to sonic stimulation of somatosensory pathways in the thalamus should be considered and addressed properly. Administration of antiemetics prior to the treatment and ensuring a slow and careful passage of the patient into and out of the magnetic resonance imaging (MRI) scanner's static magnetic field are preventive measures. The presence of an anesthesiologist to immediately treat severe nausea by providing suction of the oral cavity in case of emesis is advisable as draining the water and taking the patient out of the treatment device may take too long. Lastly, lower-back pain, camptocormia, a short neck and obesity must be considered during evaluation of patients for MRgHiFUS therapy.

#### MRI Contraindications, Implants, Scars

##### Evidence

Only few scientific evidences are available about MRgHiFUS contraindications due to local pathology. Yang et al. published a technical note and report of one patient who received MRgHiFUS thalamotomy for ET despite an implanted VP shunt. They found no adverse effects and only a small number of elements which had to be switched off to prevent damaging the shunt components (32).

##### Expert Opinion

Obviously, all contraindications for high-field MRI apply, such as MRI-incompatible medical implants, claustrophobia, inability to lay prone for extended periods of time, etc. should be thoroughly considered. In addition, scars in the scalp may lead to trapped air bubbles on the skin surface hindering the treatment. Old trepanations, craniotomies or cranioplasties must be also considered during intervention planning and frame fixation to prevent accidental heating, skin burns, off-target tissue ablation and skull fractures or impressions. For cranial implants like CSF shunts, there is limited experience available so far, although such implants, if generally MR compatible, might not necessarily be a strict contraindication. The safety of the patients should be considered extremely carefully, cases be published and unusual conditions registered in a registry.

### Targets for MRgHiFUS

*Should MRgHiFUS lesioning apply the same stereotactic targets as currently established for DBS?*

#### Evidence

There is a lack of comparative studies of DBS, RFA, MRgHiFUS, and radiosurgical ablations, but in a thorough meta-analysis of the published results of all these techniques, Dallapiazza et al. found comparable response rates and complication rates, indicating that indeed the same targets which apply for DBS may apply for MRgHiFUS (11).

#### Expert Opinion

Functionally, MRgHiFUS lesions and DBS should elicit comparable neurological effects if placed identically in spite of the different mechanisms of action and even though further studies are needed to understand better specific mechanisms of action of DBS. However, the methods differ in their flexibility to access and cover functional targets and in their risk profiles for inducing side effects. If targets are within the accessible volume dictated by ultrasound physics, MRgHiFUS theoretically can create lesions of any size or form by combining successive point lesions and therefore could paint anatomical structures for optimal target coverage. However, doing so can be very time consuming and transient edema associated with thermally induced lesions might lead to intra- and postoperative adverse effects that resolve only after days or weeks. In addition, off-target tissues could accumulate enough thermal dose during the process to get permanent thermal damage. DBS on the other hand, can adapt the geometry of the stimulation field around the electrode to optimize treatment effect and over time can follow disease dynamics. Furthermore, new DBS technique using “closed loop” modulation of the stimulation (intensity and duration) could better adapt to symptoms variability (33, 34). Still, limitations of field shaping due the specific geometry of contacts might lead to excess stimulation of surrounding tissues and might require careful balancing of beneficial and adverse stimulation effects.

#### ET

##### Evidence

The efficacy of MRgHiFUS thalamotomy of the VIM in ET was shown in randomized controlled trials (2, 14, 35). Gallay et al. reported a series of 50 cerebellothalamic tract (CTT) lesions (and several other targets and indications) with MRgHiFUS with low complication rates (36). Boutet et al. found the best effect after lesioning the most posterior portion of the Vim in a retrospective analysis of 66 cases (37) and Pineda-Pardo et al. correlated microstructural changes to the dentato-rubro-thalamic tract (equates CTT) with a good response of tremor after lesioning (38). The evidence level is Ib. The best location is probably the entry of the CTT into the thalamus, i.e., just below the VIM itself.

##### Expert Opinion

Regarding essential tremor, in principle the same targets used for DBS could be also applied for MRgHiFUS. The VIM surely represents a suitable and established target with a favorable risk profile (39). Additionally, positive experiences with the CTT in DBS were reported by members of the group and might be considered as an alternative target especially in cases of decreasing clinical effect after VIM MRgHiFUS.

#### PD

##### Evidence

Bond et al. published the results of a randomized controlled trial on MRgHiFUS thalamotomy of the VIM for tremor dominant PD in 2017, showing good tremor response and low side effects (1). The evidence level is Ib. Martínez-Fernández et al. just recently published a randomized controlled trial of unilateral STN ablations in highly asymmetrical PD with good results (evidence level Ib). So far, there is no published controlled study on pallidotomy for PD (19).

##### Expert Opinion

Aside from unilateral VIM lesioning for tremor-dominant PD, lesioning of the pallidothalamic tract (PTT), as an alternative to VIM, could lead to decreased responsiveness to L-Dopa medication. Therefore, even unilateral MRgHiFUS of that target may lead to freezing, dysarthria, and dysphagia in patients at risk.

Lesioning the GPi is difficult as it is located more lateral and closer to the outer margin of the brain volume less accessible to the MRgHiFUS system. Patients with relatively low SDR (below 0.4) might not qualify for such a treatment.

In summary, MRgHiFUS lesioning cannot be presently applied to all stereotactic targets as currently established for DBS. Only CCT and VIM can be considered as established targets and all others should be further investigated in prospective controlled clinical studies.

### Bilateral Treatments With MRgHiFUS

*Experiences with and risk evaluation for bilateral ablative treatment in terms of recommended indications and temporal staging?*

#### Evidence

Technically, MRgHiFUS allows for bilateral treatments, both within the same treatment session, or in a staged manner during two consecutive sessions. Reasons for simultaneous bilateral treatments could be to increase therapeutic effects, f. i. for neuropathic pain, or to save patients from the burden of multiple hospitalizations. Staged bilateral treatments could be considered to extend therapeutic benefit to the contralateral side or in case of axial symptoms like head or voice tremor. In addition, multiple functional targets could be applied to try regulating pathologic brain function via multiple pathways.

Current literature from RFA and DBS describes potential risks of bilateral treatments which are considered also relevant for MRgHiFUS (40). In particular, experience from earlier pallidotomy demonstrate that bilateral lesioning may harbor the risk of considerable side effects (e.g., corticobulbar syndrome, apathy, falls etc.) (37). Recent publication of a retrospective case-series of 9 patients with ET and another prospective series of 10 ET patients who received staged bilateral thalamotomies showed a good therapeutic effect on both sides with only low complication rates. Careful patient selection and exclusion of patients with pre-existing gait disorders was found to be key for the treatment success (41, 42). The evidence level is IIb.

#### Expert Opinion

If applied, treatments should be staged at least three, better even 6 months after initial MRgHiFUS and the second treatment only considered in absence of side effects from the initially treated side. Overall, bilateral treatments are still considered experimental. Risk-benefit evaluation will depend strongly on indications, targets to be assessed and symptomatic patient status.

### Standardization of Patient Evaluation for MRgHiFUS

*How should pre- and post-interventional examinations and assessments for MRgHiFUS patients be standardized?*

#### Evidence

There is no relevant data and evidence available on this topic.

#### Expert Opinion

For reasons of comparability and integration of registries pre- and post-interventional evaluations of MRgHiFUS patients should be carefully standardized using state-of-the art assessment tools and internationally accepted norms. Wherever applicable, MRgHiFUS and DBS interventions should be assessed using identical protocols except for intervention-specific data.

### Long-Term Efficacy of MRgHiFUS

*What should we expect for long-term treatment outcomes after MRgHiFUS?*

#### Evidence

MRgHiFUS lesioning is still a relatively new treatment and consequently, knowledge about long-term treatment outcome is limited. The longest follow-up data published so far demonstrated efficacy for up to 4 years (2, 5, 35, 43). Anatomically, it is well-documented that MRgHiFUS lesions created at moderate peak temperatures and moderate accumulated thermal dose can become completely invisible in MRI some 12 months post intervention. As a general rule and in strong contrast to radiation lesions, thermal MRgHiFUS lesions show their largest extension in MRI 24–48 h post intervention and from then on shrink both in T2 and in T1 imaging. However, functionally, deactivated neurons cannot be replaced, and long-term efficacy of ablative interventions will depend both on brain plasticity, that is, the capacity to recruit new neurons within damaged networks, and on the extent of progressing neurodegeneration and associated emergence of new pathologic network activity. For ET the problem of possible tolerance to DBS treatment after years is not reported so far in the case of RFA or MRgHiFUS-generated lesions (11). Long-term follow-up of tremor patients treated with MRgHiFUS ablation will be essential in the future to evaluate these two treatment concepts against each other.

#### Expert Opinion

Continued evaluation of the patients who were enclosed in RCTs to learn about long-term efficacy of MRgHiFUS treatment is one important step but will not provide generalizable data easily. The method will also have to prove its worth in comparison with the treatment gold-standard. The best way to provide the required data for these observations is to include as many cases as possible in national or even international registries. One problem still unsolved is the heterogeneity of the used endpoints in studies and registries.

## Discussion

The primary objective of the workshops and consensus meetings was to discuss in-depth the current status of MRgHiFUS and to provide guidance to those involved in MRgHiFUS therapy. Secondarily, it was aimed to evaluate the potential benefits, specific challenges, and distinctive risks of this novel technology, and to promote further clinical and scientific exchange of experience and outcome data. Finally, by establishing the Swiss MRgHiFUS Working Group, an interdisciplinary network for MRgHiFUS and future clinical and scientific collaboration was created.

This article describes issues that are relevant and of potential interest not only for MRgHiFUS experts, but also for general practitioners, neurologists and neurosurgeons and provides consensus-based recommendations in key areas of uncertainty. A number of questions currently remain unaddressed or could not be completely answered and therefore corresponding studies are highly desired. A priority for this research regards experimental indications such as chronic neuropathic pain and OCD. Carefully designed clinical studies will be required to optimally integrate these interventions into currently available treatment strategies.

It should be noted that the current recommendations were formulated by medical professionals practicing in the setting of the national Swiss healthcare system which might affect their generalizability to a broader patient population outside Switzerland. Still, Switzerland was among the first countries to introduce MRgHiFUS treatment and has already longstanding experience in that field. One more important limitation of the present consensus statement is that it does not provide a similarly high level of evidence such as a systematic review but relies on the experiences and opinions of a group of experts. The members of the Swiss MRgHiFUS workgroup think that at the given time the number of available RCTs is still too small to justify a systematic review. The consensus statement deemed to be the proper method as it added the experience of a group of experts, which is representative for Switzerland, to today's available evidence. As the number of publications on MRgHiFUS is growing exponentially, the consensus statements were outdated already when the manuscript was first drafted. To provide a timely statement, a third conference was held, which dealt with recent developments and publications.

It is expected that the evidence included in the article and consensus-based recommendations will be helpful and provide valuable guidance for clinicians and healthcare professionals involved in the treatment of patients with MRgHiFUS.

## Conclusion

These recommendations (Table 1) are based on current literature and clinical experience and aim to serve as a primer for harmonizing and optimizing patient management and best clinical practice regarding MRgHiFUS therapy in Switzerland. The vivid discussions confirmed the need for regular meetings and information exchange among the specialists in the field involved in the diagnosis and therapy of disorders treatable by functional neurosurgery. As is apparent from this manuscript, many topics need further elaboration and integration of future clinical experience.

**Table 1:** Summary of the core recommendation for the use of MRgHiFUS.

Indication	MRgHiFUS-based ablative interventions are a complement to existing functional neurosurgery treatment approaches such as DBS Essential tremor (ET) and tremor-dominant PD are presently the only indications for MRgHiFUS treatments with good level of evidence Neuropathic pain and PD with mostly unilateral motor symptoms are promising potential new indications and are under investigation
Clinical setting	MRgHiFUS treatment should only be conducted in centers where conservative and alternative surgical treatment options like DBS can be offered as well and where the therapy can be evaluated by multidisciplinary boards
Patient selection	Thorough pre-interventional assessment of motor and non-motor symptoms is mandatory MRgHiFUS should be considered in patients who present with contraindications for DBS In addition, patient selection should start focusing on individual risk-benefit evaluations between MRgHiFUS, DBS and other modalities
Procedure	To prevent unsuccessful treatment attempts, unintended off-target ablations and damage to skin and bone marrow, deep knowledge of the technical aspects of MRgHiFUS is mandatory and each patient must be evaluated thoroughly for eligibility for this kind of treatment by a multidisciplinary team
Contraindications for MRgHiFUS	Absolute: Contraindication to high field MRI Active and refractory epilepsy Anticoagulation or antiaggregation Lack of cooperation Relative: Necessity to perform under general anesthesia Low SDR Relevant medical conditions such as low back pain, camptocormia, short neck and obesity Presence of gait disorder
Targets	Unilateral VIM or CTT for ET and tremor in PD All other targets should be considered as experimental and be examined in clinical studies
Bilateral lesioning	Presently not recommended In exceptional cases, contralateral treatment should be staged at least six months after initial MRgHiFUS and the second treatment only considered in absence of side effects from the initially treated side
Long-term efficacy	Not known yet, maximal published follow-up is 4 years

It also became apparent that DBS and MRgHiFUS should not be treated in separate communities and should not be played off against each other. Rather, they should be adopted as mutually complementing therapy options that should be appreciated for their distinct advantages and therapy potential to further extend the therapeutic spectrum and optimize as well as tailor patient treatment.

## Data Availability Statement

The original contributions presented in the study are included in the article/Supplementary Material, further inquiries can be directed to the corresponding author/s.

## Author Contributions

LS and AK-L wrote, reviewed, and finalized the manuscript. All authors contributed to the workshop and to the manuscript in co-editing.

## Funding

The consensus meetings and workshops were organized and supported by the SMDS.

## Conflict of Interest

The authors declare that the research was conducted in the absence of any commercial or financial relationships that could be construed as a potential conflict of interest. The reviewer RF declared a past co-authorship with several of the authors LS, CB, FB, PK, and SM to the handling Editor.

## Publisher's Note

All claims expressed in this article are solely those of the authors and do not necessarily represent those of their affiliated organizations, or those of the publisher, the editors and the reviewers. Any product that may be evaluated in this article, or claim that may be made by its manufacturer, is not guaranteed or endorsed by the publisher.

## Organizing Committee

Lennart H. Stieglitz, Alain Kaelin-Lang, and Georg Kägi.

### Swiss MRgHiFUS Working Group

Ettore A. Accolla, Neurology Unit, Department of Internal Medicine, HFR - Cantonal Hospital Fribourg, and University of Fribourg, Fribourg, Switzerland

Julien Bally, Department of Neurology, Lausanne University Hospital, Lausanne, Switzerland

Roland Bauer, Department of Neurosurgery, Cantonal Hospital Aarau, Aarau, Switzerland

Christian R. Baumann, Department of Neurology, University Hospital Zurich, Zurich, Switzerland

David Benninger, Department of Neurology, Lausanne University Hospital, Lausanne, Switzerland

Stephan Bohlhalter, Neurocenter, Lucerne Cantonal Hospital, Lucerne, Switzerland

Fabian Büchele, Department of Neurology, University Hospital Zurich, Zurich, Switzerland

Stefan Hägele-Link, Department of Neurology, Cantonal Hospital St. Gallen, St. Gallen, Switzerland

Georg Kägi, Department of Neurology, Cantonal Hospital St. Gallen, St. Gallen, Switzerland

Alain Kaelin-Lang, Neurocenter of Southern Switzerland EOC, Regional Hospital Lugano, Lugano, Switzerland

Paul Krack, Department of Neurology, Bern University Hospital, Bern, Switzerland

Marie T. Krüger, Department of Neurosurgery, Cantonal Hospital St. Gallen, St. Gallen, Switzerland

Sujitha Mahendran, Department of Neurology, University Hospital Zurich, Zurich, Switzerland

J. Carsten Möller, Center for Neurological Rehabilitation, Zihlschlacht, Switzerland

Veit Mylius, Department of Neurology, Center for Neurorehabilitation, Valens, Switzerland

Markus F. Oertel, Department of Neurosurgery, University Hospital Zurich, Zurich, Switzerland

Tobias Piroth, Department of Neurology, Cantonal Hospital Aarau, Aarau, Switzerland

Lennart H. Stieglitz, Department of Neurosurgery, University Hospital Zurich, Zurich, Switzerland

Beat Werner, Center for MR Research, University Children's Hospital Zurich, Zurich, Switzerland.
