# Supplementary material for: Consensus Statement on High-Intensity Focused Ultrasound for Functional Neurosurgery in Switzerland
Source: Front Neurol. 2021 Sep 22;12:722762. doi: 10.3389/fneur.2021.722762 (PMC8493868; doi:10.3389/fneur.2021.722762)
Supplement: Supplementary file 1 [file Data_Sheet_1.docx]

Consensus Statement on High-Intensity Focused Ultrasound for Functional Neurosurgery in Switzerland

# Supplementary Material: Situation in Switzerland

## Registry for MRgHiFUS

*Should Swiss-wide registries for MRgHiFUS and DBS interventions be established?*

Registries are a valuable tool for assessing outcome data of medical therapies. For movement disorders, the Federal Office of Public Health (FOPH) has maintained since 2015 a registry to collect outcome data from MRgHiFUS interventions conducted at the University Hospital Zurich and at the SoniModul Solothurn.

Furthermore, the existing movement disorders registry at the Cantonal Hospital St. Gallen for MRgHiFUS and DBS interventions could serve as a platform for unified evaluation of patients with movement disorders. These registries could serve as a basis for extended collection of outcome data, i.e., for other indications than movement disorders, and other modalities than MRgHiFUS and DBS, such as drug delivery pumps. Such registries could benefit from the experiences collected for example by the Swiss Stroke Registry, or the outcome databases of the University Hospital Zurich. The first mandates for certification process implementation in the Swiss Intercantonal Agreement on Highly Specialized Medicine (IV-HSM) have been transferred to the Swiss Federation of Clinical Neuro-Societies (SFCNS), the umbrella association of all Swiss neuro-societies which is responsible for the Intercantonal Federation about IV-HSM and would therefore be the logical owner of the registry and would be a suitable body to put required contracts, logistical and ethical framework into place.

## Clinical research for MRgHiFUS

*Ideas and need for clinical studies, scientific projects and coordination of research efforts?*

Currently, significant basic research efforts are conducted at the Universities of Zurich and Geneva and the Federal Institute of Technology Zurich (ETH) to better understand the biophysics of focused ultrasound and to translate research results into clinical applications. Within the context of the SMDS, research could concentrate on evidence-based outcome measures in the form of registries and literature analysis of past and new clinical outcome data to ensure timely criteria for patient selection and treatment recommendations and highly importantly support for basic research of pathophysiological models of functional brain disorders and their translation into treatment strategies. Furthermore, the SMDS could actively engage in education and dissemination of research results into the neurofunctional clinical community to foster mutual understanding and collaboration.

## Dissemination of information and knowledge transfer

*How to ensure coherent communication about MRgHiFUS based on up-to-date technological knowhow and high-quality outcome data?*

In today’s communication culture new medical modalities face communication challenges on three distinct levels: On the public level, mass media and internet channels can very effectively address patients and patient organizations to create demand for new therapy approaches. While this can be very helpful to accelerate clinical introduction of new treatment options it comes at the risk of biased information leading to unrealistic patient expectations and frustration. The Swiss MRgHiFUS Working Group therefore considers it essential that medical professionals working with functional brain disorders, their medical societies (e.g. SMDS, Swiss Neurological Societies) and very importantly the respective patient organizations (e.g. Swiss Parkinson Association) are regularly updated on the current clinical experience with MRgHiFUS and other interventional and drug-based therapies to ensure coherent communication and understanding within the field and towards patients. On the professional level, the working group recommends embracing MRgHiFUS as a valid therapy option to DBS and drug pumps and to include it into the curricula of academic education and professional training. On a regulatory level, acceptance of MRgHiFUS as standard of care with associated reimbursement by the healthcare system will depend on solid outcome data from harmonized registries to benchmark risk-benefit/overall cost of different modalities for different indications and patient populations. While FOPH and health insurance companies have voiced interest in MRgHiFUS partially for cost reasons such claims will need clinical justification from outcome data in terms of procedural risks, quality and stability of treatment effect, patient eligibility and total cost of treatment. As a very positive step in this direction, the newly founded Intercantonal decision board of the Swiss Intercantonal Agreement on IV-HSM guidelines, ruled that MRgHiFUS will be treated similarly to DBS.
